# Patient preferences of chemotherapy treatment options and tolerance of chemotherapy side effects in advanced stage lung cancer

**DOI:** 10.1186/s12885-019-6054-x

**Published:** 2019-08-27

**Authors:** K. M. Islam, T. Anggondowati, P. E. Deviany, J. E. Ryan, A. Fetrick, D. Bagenda, M. S. Copur, A. Tolentino, I. Vaziri, H. A. McKean, S. Dunder, J. E. Gray, C. Huang, A. K. Ganti

**Affiliations:** 1 Medical College of Augusta, Augusta University, 1120 15th Street, CJ 2326, Augusta, GA 30912 USA; 2 Department of Epidemiology, University of Nebraska Medical Center College of Public Health, Omaha, NE USA; 3 Nebraska Cancer Coalition, Lincoln, NE USA; 4 Department of Health Services Research and Administration, University of Nebraska Medical Center College of Public Health, Omaha, NE USA; 5 Department of Anesthesiology, University of Nebraska Medical Center College of Medicine, Omaha, NE USA; 6 Marry Lanning Healthcare System, Hastings, NE USA; 7 Avera Cancer Institute, Sioux Falls, SD USA; 8 Saint Luke’s Hospital of Kansas City, Kansas City, MO USA; 9 Callahan Cancer Center, North Platte, NE USA; 10 Southeast Nebraska Cancer Center, Lincoln, NE USA; 11 H. Lee Moffitt Cancer Center & Research Institute, Tampa, FL USA; 12 Department of Veterans Affairs Medical Center, Kansas City, MO USA; 13 Veterans Administration Health Care Systems Medical Center, Omaha, NE USA; 14 Department of Internal Medicine, School of Medicine, University of Nebraska Medical Center Division of Oncology-Hematology, Omaha, NE USA

## Abstract

**Background:**

In the U.S., lung cancer accounts for 14% of cancer diagnoses and 28% of cancer deaths annually. Since no cure exists for advanced lung cancer, the main treatment goal is to prolong survival. Chemotherapy regimens produce side effects with different profiles. Coupling this with individual patient’s preferred side effects could result in patient-centered choices leading to better treatment outcomes. There are apparently no previous studies of or tools for assessing and utilizing patient chemotherapy preferences in clinical settings.

The long-term goal of the study was to facilitate patients’ treatment choices for advanced-stage lung cancer. A primary aim was to determine how preferences for chemotherapy side effects relate to chemotherapy choices.

**Methods:**

An observational, longitudinal, open cohort study of patients with advanced-stage non-small cell lung cancer (NSCLC) was conducted. Data sources included patient medical records and from one to three interviews per subject. Data were analyzed using Chi-square, Fisher’s Exact and McNamara’s test, and logistic regression.

**Results:**

Patients identified the top three chemotherapy side effects that they would most like to avoid: shortness of breath, bleeding, and fatigue. These side effects were similar between first and last interviews, although the rank order changed after patients experienced chemotherapy.

**Conclusions:**

Patients ranked drug side effects that they would most like to avoid. Patient-centered clinical care and patient-centered outcomes research are feasible and may be enhanced by stakeholder commitment. The study results are limited to patients with advanced NSCLC. Most of the subjects were White, since patients were drawn from the U.S. Midwest, a predominantly White population.

## Background

Lung cancer is the leading cause of cancer-related deaths in the United States (U.S.) [1]. In 2016, more than a quarter of a million new cases of lung cancer were reported [2]. In comparison to other cancers in the U.S., lung cancer, which has an average age at diagnosis of 70 years, is a major source of health care costs and utilization of health care services [3, 4].

The treatment options for non-small cell lung cancer (NSCLC) are based mainly on the stage of the cancer. Other factors, however, such as a person’s health status, lung function, and characteristics of the cancer, are also considered. Treatment goals for NSCLC are to prolong survival and control disease-related symptoms [5]. For patients who are not candidates for molecularly targeted therapy, use of various platinum doublets have led to similar survival outcomes and are recommended by current National Comprehensive Cancer Network (NCCN) guidelines [6, 7]. Furthermore, there are different toxicity profiles for the most commonly used chemotherapy drugs [8]. Therefore, toxicity profiles are involved in determining treatment choices, patient tolerability of chemotherapy, and treatment success [9].

Although most cancer patients prefer either an active or shared role in decision-making [10, 11], no definitive clinical guide on how to obtain and integrate their preferences of side effects in treatment decisions have been published, Moreover, most providers lack the tools, time, and resources to consider, efficiently and effectively, such patient-centered treatment plans [12].

The long-term goal of the present study was to determine patients’ chemotherapy treatment choices for advanced-stage lung cancer, utilizing their preferences of drug options before and after experiencing effects of treatment. An aim was to assess treatment choices of the patients based on their ranking of unwanted drug side effects. We were particularly interested in: (1) whether patients’ characteristics are associated with the length of time they are willing to tolerate chemotherapy side effects to attain a personal goal; (2) whether the length of time patients are willing to tolerate chemotherapy side effects to attain a personal goal changes after receiving chemotherapy; (3) identifying the drug side effects (and thus the drug profiles) that are least tolerable to patients; and (4) whether the ranking of drug profiles changes after patients receive chemotherapy.

## Methods

### Patients

**Fig. 1:**
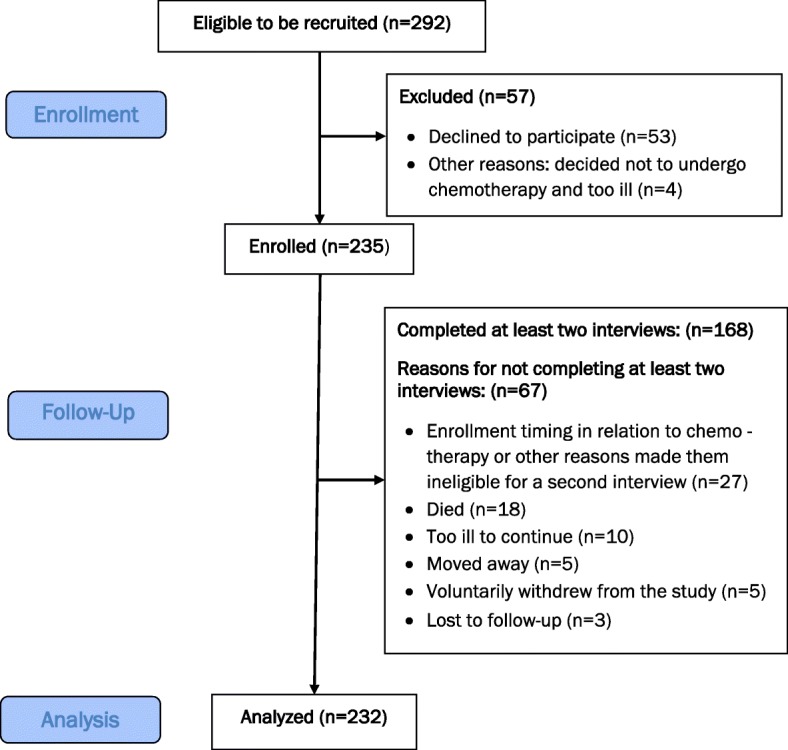
Participant Flow

A prospective, open cohort study to assess treatment choices of patients based on their ranking of unwanted drug side effects was conducted. We recruited 235 adult patients (Fig. 1 shows the detail recruitment flow chart) with advanced non-small cell lung cancer (NSCLC) from nine cancer center sites located mainly in the U.S. Midwest between January 2014 and March 2016. Eligibility criteria included patients diagnosed with advanced stage (stage 3b and above) NSCLC, age 19 years or older with the ability to understand spoken English and willing and able to provide informed consent, and who were eligible to undergo chemotherapy for advanced stage NSCLC.

Participants were followed by site staff who collected medical record and interview questionnaire data before, during, and after first-line chemotherapy. Each participant had at least one interview, and 71% (168/235) had at least two interviews. The median follow-up of the entire group (including those with one interview) was 1 month and 6 days (Range 0–13 months). In addition to documenting the details of their chemotherapy treatment experience via reviews of medical records and personal interviews, preferences of patients and the ranking of side effects were sought during scripted interviews before and after chemotherapy. This information was used to identify the chemotherapy drugs that were likely to produce the side effects patients most wanted to avoid.

A sample size of 210 patients at baseline will produce a 95% confidence interval equal to the sample proportion plus or minus 5% (PASS 2005; NCSS LLC; Kaysville, Utah). We assumed to 10–15% patient will miss the follow up interview for various reasons including death. To compensate the loss to follow up, we recruited 235 patient for this study.

### Outcomes, measures, and data collection

Our primary aim was to determine how preferences for chemotherapy side effects relate to chemotherapy choices. Therefore, the outcomes included data regarding patient preferences and tolerance levels regarding chemotherapy treatment, specifically, the length of time they were willing to tolerate side effects, which side effects they would most like to avoid (which reveals which drug profiles are most and least tolerable), and whether or not preferences and tolerance levels change based on experience with chemotherapy. To measure the length of time that patients were willing to tolerate the side effects, they responded to a question with three categories of timelines related to length of tolerance (no time, less than 12 months, more than 12 months) as part of a questionnaire administered by an interviewer or completed by the subject (nearly all chose to be interviewed). Research staff conducted interviews or arranged for questionnaires to be completed before chemotherapy treatments began, after one or a few treatments had been administered, and/or nearing the end of first-line treatment as appropriate to the circumstances of the patients.

To rank the side effects, we generated an inventory based on those reported to be associated with chemotherapy and found on the http://www.uptodate.com website. From these data, we developed a table with the adverse events frequently reported for chemotherapy drugs commonly used for treating NSCLC. Four drugs were chosen because they were among the most-frequently administered drugs for advanced-stage NSCLC and because their adverse side effects profiles were different enough to distinguish them from others. We identified drugs that: (1) are typically used for first-line chemotherapy treatment of advanced stage NSCLC, (2) had discriminatory profiles for adverse side effects, and (3) had side effects that could be recognized by most patients. The four drugs that best fit these criteria required the use of nine side effects to identify patient preferences and tolerance levels that could give the physician actionable information.

Based on our inventory of adverse side effects and profiles of the four drugs, we developed a survey tool, the ‘Ranking Exercise,’ to collect preferences of patients and their estimated tolerability levels of side effects they would most like to avoid (Table 1). The nine discriminatory side effects were, in alphabetical order: (1) excessive *bleeding*, (2) shortness of *breath*, (3) *brittle* nails, (4) *dizziness*, (5) considerably more *expensive* than other chemotherapy, (6) excessive *fatigue*, (7) *numbness* and/or tingling, (8) more *trips* to the clinic for chemotherapy, and (9) *yellow* skin/ jaundice. Recognizing that patients would like to avoid all adverse side effects of chemotherapy drugs, we asked them to tell us which ones they would *most* like to avoid. Participants rank-ordered side effects from one to nine, with those they would most like to avoid called, ‘bad,’ and given a number “1” (first rank) and those that they thought were most tolerable, ‘least bad,’ and given a number “9” (ninth rank), with the other seven side effects given ranking positions “2” through “8.” Subjects indicated their preferences and tolerance levels for the nine discriminatory side effects.

**Table 1:** Ranking exercise

Possible Side Effects	Rank Order 1 to 9 Bad to Least Bad
A. brittle nails	A =
B. decreased energy (excessive fatigue)	B =
C. dizziness	C =
D. unusual / increased bleeding	D =
E. jaundice (yellow skin)	E =
F. more trips to clinic for treatment	F =
G. numbness and / or tingling	G =
H. shortness of breath	H =
I. a lot more expensive	I =

We linked the side effect that the patient indicated they would most like to avoid with the side effects profile of at least one drug. Since a side effect could be associated with more than one drug, and any drug might be associated with more than one side effect, we weighted the side effects based on the proportion of adverse side effects for each drug and prepared an algorithm that discriminated between the four chemotherapy drugs based on the ranking positions given by the patient and the types and frequencies of adverse side effects reported. Therefore, we could identify which drug(s) patients would most like to avoid and labeled these Drugs A, B, C, and D. Although this exercise can be accomplished with each of the other eight side effects for each patient, in this report, we concentrate on the simple iteration that allowed us to link the top side effects that the patient would most like to avoid with the drug profiles that are most highly associated with the reported adverse effects, facts that can be ascertained by physicians and others in a clinical setting.

### Analytical and statistical approaches

Data were tabulated to describe the proportional distribution of the ‘length of time’ outcome variable categorized as ‘no time period,’ a time less than 1 year (‘months’), and time more than 1 year (‘years’). In addition, we described proportions of drug side effects based on ranking by patients before and after chemotherapy and linked the drug that was connected to the least preferred side effect.

Chi-square or Fisher’s exact test was used, as appropriate, to examine the association between each patient’s characteristics and outcomes, separately. To meet the assumption of paired data, McNamara or Bowker’s test was used to assess the discordance of individual patients’ responses between first and last interviews. Chi-square or Fisher’s exact test was used to examine the association between patients’ characteristics and the concordance between drugs to avoid and drugs to receive. The significance level for all analyses was set at *p*-value < 0.05. All statistical analyses were performed using the statistical software package SAS, version 9.4 (SAS Institute Inc., Cary, NC).

Per study protocol, we had planned to employ multiple imputation only if the missing data proportion was greater than 10%. Since the highest missing data proportion of variables used in our analysis was 7.8%, we analyzed the data by excluding missing values and did not use validated methods to deal with missing data because according to statistical standards, this low level of missing data is unlikely to impact data estimates negatively.

## Results

None of the patient characteristics we tested showed statistical significance associated with the period that patients were willing to tolerate drug side effects. The results were consistent between the first and last interviews. Interviews that occurred at enrollment were termed ‘before’ or ‘first interviews’ and those that occurred after patients had more chemotherapy, were called, ‘after’ or ‘last’ interviews. Although not statistically significant, marital status showed a borderline association, *n* = 232; *p* = 0.059 in the first interview, and *n* = 167; *p* = 0.078 in the last interview (Tables 2 and 3). A higher proportion of married patients were willing to tolerate chemotherapy for months or years relative to unmarried patients, who tended not to be willing to tolerate side effects of chemotherapy treatment for any period (not shown).

**Table 2:** Tolerance time at FIRST interview by patients’ characteristics (*n* = 232)^a^

Variable	Category	Tolerance time at FIRST interview (*n* = 232)^a^			Total n (%)	*P*-value
		No time period (*n* = 36) n (%)	Months(*n* = 96) n (%)	Years(*n* = 100) n (%)		
Age group (years)	≤ 60 years	9 (25.0)	20 (20.8)	22 (22.0)	51 (21.7)	0.9788
	61–70 years	12 (33.3)	37 (38.5)	36 (36.0)	85 (36.2)	
	> 70 years	15 (41.7)	39 (40.6)	42 (42.0)	96 (41.4)	
Gender	Male	24 (66.7)	55 (57.3)	50 (50.0)	129 (55.6)	0.2052
	Female	12 (33.3)	41 (42.7)	50 (50.0)	103 (44.4)	
Race	White or Caucasian	33 (91.7)	93 (96.9)	95 (95.0)	221 (95.3)	0.5415**
	Black/African-American	2 (5.6)	2 (2.1)	2 (2.0)	6 (2.6)	
	Other	1 (2.8)	1 (1.0)	3 (3.0)	5 (2.2)	
Education^a^	Less than high school or high school diploma or GED degree	21 (61.8)	49 (51.6)	49 (50.5)	119 (52.7)	0.5081
	Some college or bachelor’s or higher degree	13 (38.2)	46 (48.4)	48 (49.5)	107 (47.3)	
Employment	Working	10 (27.8)	25 (26.0)	25 (25.0)	60 (25.9)	0.9468
	Not working	26 (72.2)	71 (74.0)	75 (75.0)	172 (74.1)	
Marital status^a^	Married	19 (52.8)	68 (71.6)	58 (58.0)	145 (62.8)	0.0588
	Not married	17 (47.2)	27 (28.4)	42 (42.0)	86 (37.2)	
Income^a^	Annual income $45,000 or more	10 (41.7)	34 (50.0)	24 (40.0)	68 (44.7)	0.4971
	Annual income less than $45,000	14 (58.3)	34 (50.0)	36 (60.0)	84 (55.3)	
Primary method of payment	Private insurance	9 (25.0)	33 (34.4)	32 (32.0)	74 (31.9)	0.1993
	Medicare	20 (55.6)	50 (52.1)	48 (48.0)	118 (50.9)	
	Medicaid	3 (8.3)	11 (11.5)	8 (8.0)	22 (9.5)	
	Others	4 (11.1)	2 (2.1)	12 (12.0)	18 (7.8)	
Urban/Rural	Urban	25 (69.4)	66 (68.8)	62 (62.0)	153 (65.9)	0.5418
	Rural	11 (30.6)	30 (31.2)	38 (38.0)	79 (34.1)	

**Uses Fisher’s Exact Test

^a^Excludes cases where values were not reported (*n* = 1, for Marital status; *n* = 2 for Income)

**Table 3:** Tolerance time at LAST interview by patients’ characteristics (*n* = 167)^a^

Variable	Category	Tolerance time at LAST interview (*n* = 167)^a^			*P*-value
		No time period(*n* = 23) n (%)	Months(*n* = 84) n (%)	Years(*n* = 60) n (%)	
Age group (years)	≤ 60 years	5 (21.7)	16 (19.0)	17 (28.3)	0.5219
	61–70 years	7 (30.4)	33 (39.3)	24 (40.0)	
	> 70 years	11 (47.8)	35 (41.7)	19 (31.7)	
Gender	Male	14 (60.9)	53 (63.1)	30 (50.0)	0.2794
	Female	9 (39.1)	31 (36.9)	30 (50.0)	
Race	White or Caucasian	21 (91.4)	81 (96.4)	58 (96.6)	0.6112**
	Black/African-American	1 (4.4)	1 (1.2)	1 (1.7)	
	Other	1 (4.4)	2 (2.4)	1 (1.7)	
Education	Less than high school or high school diploma or GED degree	15 (65.2)	47 (58.0)	27 (45.8)	0.1932
	Some college or bachelor’s or higher degree	8 (34.8)	34 (42.0)	32 (54.2)	
Employment	Working	7 (30.4)	25 (29.8)	15 (25.0)	0.7939
	Not working	16 (69.6)	59 (70.2)	45 (75.0)	
Marital status	Married	12 (52.2)	63 (75.0)	38 (63.3)	0.0780
	Not married	11 (47.8)	21 (25.0)	22 (36.7)	
Income	Annual income $45,000 or more	6 (42.9)	32 (50.8)	16 (40.0)	0.5443
	Annual income less than $45,000	8 (57.1)	31 (49.2)	24 (60.0)	
Primary method of payment	Private insurance	8 (34.8)	31 (36.9)	22 (36.7)	0.6061
	Medicare	13 (56.5)	37 (44.1)	24 (40.0)	
	Medicaid	0 (0.0)	9 (10.7)	9 (15.0)	
	Others	2 (8.7)	7 (8.3)	5 (8.3)	
Urban/Rural	Urban	15 (65.2)	59 (70.2)	40 (66.7)	0.8520
	Rural	8 (34.8)	25 (29.8)	20 (33.3)	

**Uses Fisher’s Exact Test

^a^Excludes cases where values were not reported (*n* = 1)

At enrollment, which was their first interview (*n* = 232), the proportion of patients who answered ‘months’ (41%) was similar to those who answered ‘years’ (43%); 16% were not willing to tolerate the side effects for any time period. After more experience with chemotherapy (*n* = 167), a higher proportion (50%) of patients indicated a tolerability for side effects for a shorter amount of time, as indicated by tolerance level responses of ‘months’ increasing to 50%, with a corresponding tolerability response of ‘years’ decreasing to 36%. About 48% of the patients (*n* = 167) changed their indication as to the length of tolerability of side effects between their first and last interviews (Table 4).

**Table 4:** Length of time patients willing to tolerate side effects

	Variable	Category (*n* = 232)^a^	n (%)
FIRST Interview	Tolerance time	No time period	36 (15.5)
		Months	96 (41.4)
		Years	100 (43.1)
	Variable	Category(*n* = 167)^b^	n (%)
LAST Interview	Tolerance time	No time period	23 (13.8)
		Months	84 (50.3)
		Years	60 (35.9)
	Variable	Category(*n* = 167)^b^	n (%)
	Change in tolerancetime between FIRSTand LAST interview	Yes	80 (47.9)
		No	87 (52.1)

^a^Excludes missing values (*n* = 3)

^b^Excludes those who did not complete at least 2 interviews and 1 missing value

Comparison of changes between first and last interviews on side effect tolerance showed that a higher proportion of patients who had at least two interviews (*n* = 167), that is, who had more chemotherapy experience, shifted their tolerance level estimate from a longer to a shorter time period: ‘years’ to ‘months,’ versus those who changed from a shorter to a longer time period: ‘months’ to ‘years.’ Among those who initially answered ‘years,’ 36% changed their answer to ‘months;’ 24% who initially answered ‘months’ changed to ‘years’ (Table 5).

**Table 5:** Changes in tolerance time between FIRST and LAST interview (*n* = 167)^a^

FIRSTinterview ↓	← LAST interview →			*P*-value**
	No time period *n* = 23↓(% of 167)	Months *n* = 84↓(% of 167)	Years *n* = 60↓(% of 167)	
No time period	5 (20.0)	13 (52.0)	7 (28.0)	0.4751
Months	9 (12.5)	46 (63.9)	17 (23.6)	
Years	9 (12.9)	25 (35.7)	36 (51.4)	

**Uses Bowker’s Test

^a^Excludes 1 missing value

As described above, patients were asked to rank discriminatory side effects associated with four commonly used chemotherapy drugs in the treatment of advanced metastatic NSCLC. Side effects that patients said they would most like to avoid were called ‘worst’ or ‘worst-ranked.’ The top three side effects that subjects would most like to avoid, shortness of breath, bleeding, and fatigue, remained the same between first and last interviews, but the order changed to fatigue, shortness of breath, and bleeding (Table 6). The worst-ranked side effect for each individual was linked with one of four chemotherapy drugs commonly used for advanced NSCLC. Drugs A and B had side effect profiles that matched nearly one-third or more of the patient preferences and tolerance data regarding which side effects they would most like to avoid. This distribution of the drugs to avoid did not change between the first and last interviews (Table 7).

**Table 6:** Proportion of patients ranked side effect that they would most like to avoid (*n* = 168)^a^

Worst-ranked side effect	FIRST Interview (%)	LAST Interview (%)
Shortness of breath	28.7	20.8
Bleeding	20.9	14.3
Fatigue	11.9	25.6
Dizziness	10.8	10.7
A lot more expensive	8.4	9.5
Jaundice	8.4	7.7
More trips to clinic for treatment	7.2	7.1
Numbness/tingling	1.8	1.2
Brittle nails	1.8	2.9

^a^Excludes those who did not complete at least 2 interviews

**Table 7:** Comparison of drug to avoid based on match between drug’s side effect profile and patients’ ranking^a^

Drug to avoid	FIRST Interview *n* = 167^b^ (%)	LAST Interview *n* = 166^b^ (%)
Drug A	56 (33.5)	42 (25.3)
Drug B	71 (42.5)	82 (49.4)
Drug C	26 (15.6)	25 (15.1)
Drug D	14 (8.4)	17 (10.2)

^a^excludes those who did not complete at least 2 interviews

^b^excludes those who did not complete the Ranking Exercise section

## Discussion

We utilized a multicenter, prospective, longitudinal, patient-centered research study to explore chemotherapy drug treatment choices for patients diagnosed with advanced NSCLC. To our knowledge, there have been no systematic studies that assess patient preferences in relation to chemotherapy drug treatment choices at the time of treatment planning or for monitoring patient-preference-based tolerance of chemotherapy for advanced-stage lung cancer.

Although there are reports on chemotherapy-related adverse side effects, treatment difficulties concerning side effects, and increased treatment cost due to management of side effects, there is apparently none that examined patients’ preferences regarding chemotherapy-related side effects. When we assessed treatment choices of patients based on their ranking of unwanted drug side effects, the results revealed that patients who were married were more willing to tolerate treatment side effects for longer periods of time than those who were not married. Perhaps the willingness of married patients to tolerate these side effects is because they wish to avoid leaving a spouse alone in case of their demise. An alternative possibility relates to the support provided by a spouse. In either case, our findings indicate that familial factors and the involvement of a spouse in the development of a treatment plan may help in ensuring adherence to the plan and higher levels of tolerability of side effects.

Between the first and last interviews, about half of the participants changed their indication as to the length of tolerability of side effects, with a large proportion of them redefining their level of tolerance in months versus years. This emphasizes the importance of clinicians re-evaluating a treatment plan using a patient-centered approach throughout the course of the treatment.

The top three side effects that patients would most like to avoid, shortness of breath, bleeding, and fatigue, remained stable between their first and last interview, after more experience with chemotherapy. However, fatigue was elevated in prominence, pushing shortness of breath and excessive bleeding to the number two and number three. One reason for these changes may have been the actual side effects experienced by the patients while going through chemotherapy. For instance, fatigue is a common side effect of cytotoxic chemotherapy. Hence, patients who experienced fatigue may have been more likely to want to avoid fatigue when questioned after their chemotherapy experience. Because fatigue is a subjective experience, patients who have not gone through chemotherapy treatment may not have clear idea about how troubling fatigue is. Strategies that monitor and try to control the effects of fatigue during chemotherapy appear to be warranted.

Two of the four drugs included at least one-third of the side effects, showing that patients would most like to avoid using them if possible. Most of the patients did not receive those drugs whose side effects they were trying to avoid (table not shown). However, a higher proportion of patients with a risk profile indicative of poorer economic and social support (i.e., with no more than a high school education, single, on Medicaid, and living in rural areas) received drugs whose drug side effects they would rather have avoided. Whether or not this observation relates to their lower ability to bargain or to less effective provider-client communication is not clear; this point needs further investigation. The findings indicate a need for clinicians to be cognizant of this particular group and to be proactive in discussing their treatment plan and the options and possibilities that are available. For all patients, our findings are helpful in highlighting the importance of incorporating their views throughout treatment as a way of improving patient-clinician communication and implementing more patient-centeredness into clinical care. The results indicate that many patients could benefit from clinical care tailored to their characteristics and preferences.

In making treatment decisions, patients consider toxicity [13], as outlined by the National Cancer Institute [3]. When faced with two chemotherapy regimens with similar efficacy, most NSCLC patients are willing to consider their side effects [14]. The present study confirmed the importance of toxicities in treatment planning, from the perspective of patients and oncologists, and indicated that the perception of patients about chemotherapy side effects changes over the course of treatment.

Previous studies of patients’ preferences for chemotherapy for NSCLC found that baseline and treatment-related characteristics are not predictive of their individual preferences regarding chemotherapy, as has been suggested for other cancers [15]. The present study corroborated these findings in the case of NSCLC and indicated that age, gender, and marital status of patients influence their definition of treatment success. The results point to the need for patient-provider communication to allow decisions to be made that are congruent with and respectful of patient’s values and circumstances.

There is limited research involving patients with advanced-stage lung cancer for examination of their involvement in making treatment decisions [16]. However, there is evidence that patients are confident in their role in clinical decision-making and that their confidence can be improved by involving them early in treatment planning [17]. Only half of cancer patients undergoing chemotherapy and/or radiation therapy perceive that they are offered treatment choices [18]. The present results show the feasibility, effectiveness, and importance of utilizing a patient-centered approach to engage patients by enrolling them in an study of something that affects them and to engage them in improving study design, execution, translation, and dissemination of the results.

Preferences of patients for treatment reflect their values, their understanding of their illness, and their understanding of the risks and benefits associated with treatment choices. Their participation in treatment decision-making is more appropriate than giving them information and choices. We developed patient-centered tools for the clinicians (ranking exercise and distress scale) to identify patients’ preferences for incorporation in the treatment plan. Our data and tools will help patients and their caregivers make informed treatment choices for the care of lung cancer. This research corroborates the statement by Barry and Edgman-Leviant that “shared decision-making is the pinnacle of patient-centered care” [19].

Although there is a potential for selection bias due to voluntary participation of the patients, its impact on the study is likely minimal since we recruited more than 80% of the patients invited to enroll. Thus, our results should be generalizable to advanced-stage lung cancer patients with characteristics similar to those in our study, which included mostly patients residing in the Midwestern area of the U.S. Another potential limitation is that this study was conducted before the approval of checkpoint inhibitors for first-line treatment of advanced NSCLC; however, the findings are still relevant for those patients who are not candidates for up-front immunotherapy.

The study findings can be used to improve patient care by enhancing physician-patient communication, screening patients for comorbidity, identifying patient preferences for chemotherapy side effects by using our patient-centered tools, monitoring and taking appropriate actions to ameliorate the effects of adverse side effects, and conducting further patient-centered clinical research. The study results, which support patient-centered cancer care, are available to clinicians and to patients and their caregivers. Further, the fact that the clinicians involved in our study were willing to incorporate patients’ preferences into their treatment plan adds to the current knowledge base in how to improve patient-centered clinical care.

## Conclusions

Our patient-centered outcomes study describes the feasibility of linking patient- supplied preference and tolerance levels information about side effects patients would most like to avoid with available drug choices and including this information in treatment planning and implementation. Conclusions of the study include that patients’ characteristics were not significantly associated with the period that they were willing to tolerate chemotherapy drug side effects and that nearly half (48%) changed their indication as to the length of tolerating side effects between their first interview and their last interview. In addition, patients were willing and capable of ranking nine discriminatory drug side effects to identify which side effects they would most like to avoid. Thus, clinicians could use this information in creating and implementing a patient-informed plan for chemotherapy treatment. We demonstrated how to link the patient-supplied preference information to specific profiles of commonly used chemotherapy drugs for the treatment of advanced-stage NSCLC. With the study results, clinicians may create and implement, and re-evaluate and adjust, a more patient-centered treatment plan using patient-derived communication throughout the course of their clinical care.

## Data Availability

The datasets generated and/or analyzed during the current study are not publicly available due patients’ confidentiality but are available from the corresponding author on reasonable request.

## Abbreviation

NSCLC: Non-small cell lung cancer
